# Spondyloarthritis Associated With Collagenous Colitis—Case Report and Literature Review

**DOI:** 10.1002/ccr3.72580

**Published:** 2026-04-24

**Authors:** Charlotte Bouvy, Abeline Kapuczinski, Sophiane Ibrahimi, Marc Léon, Nathalie Demeulenaere

**Affiliations:** ^1^ Rheumatology Department CHU Helora Site Kennedy Mons Belgium; ^2^ Rheumatology Department Centre Hospitalier Epicura Baudour Belgium; ^3^ Gastroenterology Department Centre Hospitalier Epicura Baudour Belgium

**Keywords:** autoimmunity, collagenous colitis, psoriatic arthritis, spondyloarthropathy

## Abstract

Collagenous colitis (CC) is a subtype of microscopic colitis characterized by chronic watery diarrhea with normal endoscopic findings. Although often linked to autoimmune diseases, its association with spondyloarthropathy (SpA) is rare and poorly documented. A 33‐year‐old man with longstanding diarrhea and polyarthralgia was initially misdiagnosed with irritable bowel syndrome and fibromyalgia. After developing palmoplantar pustulosis and worsening musculoskeletal pain, repeat colonoscopy with biopsies confirmed CC. Imaging revealed sternal erosions and metatarsophalangeal effusions, supporting a diagnosis of SpA. Treatment with budesonide, mesalazine, and methotrexate provided partial improvement. A TNF‐α inhibitor is being considered to target both intestinal and joint involvement. This case underscores the diagnostic complexity of CC and its potential association with SpA. Shared immune mechanisms suggest a common pathogenesis, highlighting the importance of multidisciplinary management for optimal care.

## Key Clinical Message

We report the case of a 33‐year‐old patient with HLA‐B27 negative axial and peripheral spondyloarthritis associated with collagenous colitis. This rare and scarcely reported association highlights the presence of shared immunopathogenic mechanisms and the link with autoimmune comorbidities. It also poses diagnostic and therapeutic challenges, requiring an integrated approach.

## 1 Introduction

Collagenous colitis (CC) is a clinicoanatomical entity first described in 1976 by Lindström. Initially considered exceedingly rare, its recognition has increased over time owing to improved understanding of the disease and advances in diagnostic methods, although its etiopathogenesis remains unclear [1, 2, 3, 4]. CC has also been linked to a variety of autoimmune disorders [5, 6, 7, 8]. In contrast, its association with spondyloarthropathy (SpA) is rare [9, 10, 11, 12]. Although the literature on CC is relatively extensive, reports focusing on articular manifestations remain limited. We report the case of a patient presenting with axial and peripheral SpA occurring in the setting of CC.

## 2 Case Description

### 2.1 Case History

A 33‐year‐old man without significant past medical history presented with diffuse polyarthralgia evolving since late adolescence. He reported pain in the lower back, neck, shoulders, wrists, hands, knees, and feet, with morning stiffness lasting up to 1 h. Pain improved with nonsteroidal anti‐inflammatory drugs (NSAIDs). Additional complaints included fatigue and sleep disturbances. Although he had no personal history of psoriasis, his father had the condition. From his early 20s, he also experienced chronic abdominal pain and diarrhea, including nocturnal symptoms and fecal urgency, without hematochezia. Prior gastroenterological evaluation had excluded inflammatory bowel disease (IBD) and celiac disease, with normal laboratory tests, negative anti‐ *Saccharomyces cerevisiae* antibodies, normal fecal calprotectin, bile acid malabsorption testing, and an unremarkable left‐sided colonoscopy. An initial diagnosis of irritable bowel syndrome (IBS) was therefore made.

### 2.2 Investigations and Treatment

The initial rheumatologic evaluation revealed no clinical arthritis or enthesitis. Laboratory tests were unremarkable: normal blood count, preserved liver and kidney function, absence of inflammatory syndrome, and negative rheumatoid factor, anticitrullinated peptide antibodies, antinuclear antibodies (ANA), and HLA‐B27. Imaging studies—sacroiliac and spinal MRI, bone scintigraphy, and radiographs of the hands and feet—were normal. A diagnosis of fibromyalgia was considered, and NSAIDs with physiotherapy were initiated. Subsequently, the patient developed a palmoplantar pustular eruption suggestive of SAPHO syndrome. Ultrasound of the hands and feet demonstrated mild joint effusions and calcific Achilles enthesopathy, while MRI of the hands remained unremarkable. Methotrexate was proposed but declined by the patient. Sulfasalazine was introduced, resulting in partial improvement of digestive symptoms but minimal change in joint pain.

**FIGURE 1:**
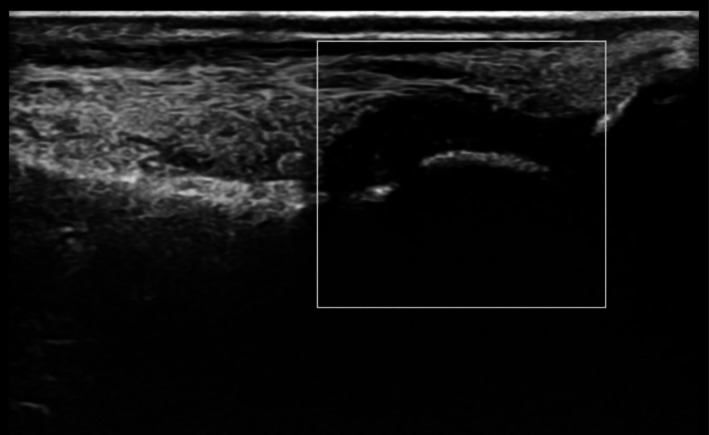
Power Doppler ultrasound of the second metatarsophalangeal (MTP) joints. Longitudinal view revealed significant synovial thickening and joint effusions, consistent with peripheral spondyloarthritis.

**FIGURE 2:**
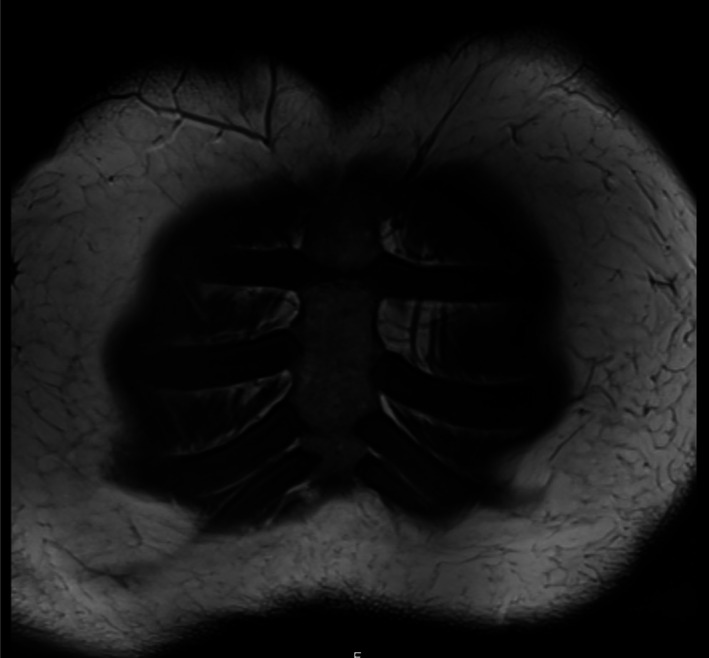
Sagittal T1‐weighted magnetic resonance imaging (MRI) of the sternum. Structural erosions are visible at the margins of the chondrosternal joints. These chronic inflammatory changes support the diagnosis of axial spondyloarthritis in the context of CC. Note the absence of significant bone marrow edema or surrounding soft tissue swelling.

A repeat colonoscopy with biopsies revealed inflammatory infiltrates of the lamina propria, thickening of the basement membrane, and lymphoid hyperplasia, consistent with CC. Combination therapy with budesonide and mesalazine improved gastrointestinal symptoms. Concurrently, the patient developed sternal pain and worsening peripheral pain involving the ankles and toes. Ultrasound revealed moderate‐to‐severe effusions in bilateral metatarsophalangeal joints (Figure 1). MRI of the sternum demonstrated erosions at the chondrosternal joint margins, without bone marrow edema or effusion (Figure 2). A diagnosis of spondyloarthritis with both axial and peripheral involvement was established. Methotrexate therapy was initiated, resulting in progressive reduction of peripheral effusions. NSAIDs were discontinued due to the diagnosis of CC.

### 2.3 Outcome and Follow‐Up

The therapeutic journey reached a clinical crossroads following NSAIDs withdrawal. Although methotrexate and budesonide partially controlled peripheral joint effusions and diarrhea, the patient continued to experience debilitating axial inflammatory pain. Given the risk of gastrointestinal flares with NSAIDs, a TNF‐α inhibitor was recommended for integrated management of both gastrointestinal and osteoarticular manifestations. Currently, the patient is in a period of therapeutic reflection, expressing hesitancy regarding biologic therapy. This case underscores the challenge of managing spondyloarthritis when first‐line therapies are contraindicated by coexisting microscopic colitis, requiring a patient‐centered approach to balance disease activity and treatment acceptability.

## 3 Discussion

### 3.1 Epidemiology and Clinical Presentation

CC belongs to the group of microscopic colitides (MC), characterized by nonbloody watery diarrhea and a macroscopically normal or near‐normal colonic mucosa on endoscopy [1, 3, 4, 13, 14]. Histologically, two main subtypes are described: lymphocytic colitis (LC) and CC [1, 2]. MC is defined by diffuse inflammation of the lamina propria with prominent intraepithelial lymphocytic infiltration, and in the case of CC, by a thickened subepithelial collagen band [15, 16]. A third entity, incomplete MC, is characterized by intermediate histopathological abnormalities [4].

Although classified among the IBDs, CC differs significantly from Crohn's disease (CD) and ulcerative colitis (UC) [1, 3, 8]. CD and UC are well known for their various extraintestinal manifestations, which are generally absent in CC. In contrast, CC is particularly noted for its high frequency of associated autoimmune disorders [5, 6, 7]. Unlike the classic IBD forms, CC has not been linked to an increased cancer risk, and some studies even suggest a protective effect against colorectal carcinoma [8].

The incidence of CC is estimated at between 2 and 10.8 per 100,000 persons per year, with higher rates in Northern Europe and North America [4, 14, 17]. The mean age at diagnosis is 65 years; about one‐fourth of patients are diagnosed before the age of 45 [3]. The female‐to‐male ratio is approximately 3:2 [4, 17]. In recent years, the incidence of CC has increased markedly, reflecting drug‐induced cases and improvements in diagnostic techniques [3, 4, 13, 18].

Clinically, CC presents with chronic, watery, nonbloody diarrhea [1, 2, 15], often occurring at night or early in the morning, accompanied by nausea, abdominal pain, bloating, fecal incontinence, and weight loss [1, 14]. In most cases, symptoms develop gradually, but approximately 40% of patients experience a sudden onset [3, 13]. Symptoms are often intermittent, although some patients exhibit a chronic, persistent course without remission, resulting in a markedly impaired quality of life (QOL) [13, 15, 16]. CC is rarely associated with severe complications, although spontaneous or postcolonoscopy perforations have been reported [8].

### 3.2 Diagnosis

The diagnosis is often challenging and requires the exclusion of other causes of chronic diarrhea [15]; many patients with MC are misdiagnosed with IBS [1, 13, 15]. Laboratory testing should include stool cultures to rule out bacterial pathogens such as *Campylobacter*, *Shigella*, *Salmonella*, *Yersinia*, *Clostridioides difficile*, and *Escherichia coli* , as well as parasitic infections [1, 3]. Celiac serologies are also recommended [15]. There are no biomarkers or specific laboratory tests for CC. CRP, ESR, and autoimmune profiles are typically normal, and radiologic imaging is unremarkable [1, 2, 3]. Fecal lactoferrin and calprotectin may be elevated but are not reliable for diagnosis or follow‐up of CC [1, 4]. Indeed, while calprotectin levels tend to be higher in active CC, up to 38% of symptomatic patients show normal values [1, 5].

MC ultimately accounts for up to 15% of cases of chronic diarrhea [4, 13, 14]. In patients with a compatible clinical picture, a colonoscopy with histological examination of multiple biopsies is required [1, 2, 14, 15]. Endoscopically, the mucosa typically appears normal, though erythema, edema, vascular pattern alterations, mucosal tears, and nodularity have been described [15, 16]. Diagnosis relies on specific histological features seen in colonic mucosal biopsies [1, 2]. Common findings in MC include irregular cuboidal surface colonocytes, reduced goblet cells, and the hallmark feature: increased intraepithelial lymphocytes [15, 16]. The lamina propria shows enhanced cellularity composed mainly of plasma cells and neutrophils, often with mild cryptitis [16]. In CC, but not LC, there is a thickened subepithelial collagen band [1, 16]. According to European consensus diagnostic guidelines, the collagen band thickness must exceed 10 μm (normal < 3 μm) in well‐oriented biopsies. There is no direct correlation between band thickness and symptom severity [1, 4].

### 3.3 Pathophysiology and Immune‐Mediated Mechanisms

CC is a multifactorial condition with an incompletely understood etiopathogenesis, likely driven by an aberrant immune response to luminal, dietary, or bacterial antigens in genetically predisposed individuals, resulting in epithelial barrier dysfunction and local inflammatory activation [11, 12, 14, 16]. The ensuing subepithelial collagen deposition reflects disturbed extracellular matrix metabolism, favored by procollagen‐I overexpression, elevated tissue inhibitors of metalloproteinases, and reduced fibrinolytic activity [12, 14, 16]. Notably, these deposits may regress with treatment [4, 10].

Genetic predisposition is suggested by the association of HLA haplotypes, notably HLA‐DQ2 and HLA‐A2, which are also found in other autoimmune diseases such as celiac disease, autoimmune thyroiditis, and type 1 diabetes [5, 6, 7]. However, no genetic marker has yet been identified [5].

CC is now recognized as an immune‐mediated disease, resulting from complex interactions between immune cells, cytokines, and the colonic epithelium [5, 6, 7, 11]. Histological analysis demonstrates diffuse lymphocytic infiltration, characterized by a predominance of CD4+ lymphocytes in the lamina propria and intraepithelial CD8+ lymphocytes [5, 6]. These cells play a key role in direct epithelial cell injury, leading to impaired mucosal barrier integrity [5, 6]. Concurrently, overexpression of HLA‐DR molecules by colonic epithelial cells has been reported, promoting antigen presentation and activation of cytotoxic T‐cell responses. On a molecular level, a proinflammatory cytokine profile has been identified, marked by overexpression of IFN‐γ, TNF‐α, IL‐1β, and IL‐6, along with increased production of nitric oxide and prostaglandins [1, 5, 6]. This inflammatory cascade contributes to the maintenance of chronic inflammation and persistent watery diarrhea [1, 2, 16].

### 3.4 Environmental Risk Factors and Drug‐Induced Colitis

Several environmental and drug‐related risk factors have been identified [3, 4, 13, 18]. Smoking is a well‐documented risk factor: smokers develop the disease on average 10 years earlier than nonsmokers, with a three‐ to fourfold increased relative risk [4, 17]. Proposed mechanisms include modification of gut microbiota composition, increased TGF‐β production, and excessive CD8+ T‐cell activation [7, 11]. Alcohol also contributes by promoting oxidative stress, disruption of tight junctions, and increased intestinal permeability [5, 7]. CC may also occur as a sequela of infectious colitis, with cases reported following *Yersinia enterocolitica* and *C. difficile* infections. Symptom resolution after *Helicobacter pylori* treatment has additionally been reported [1, 16].

Among drugs, NSAIDs are most frequently implicated through prostaglandin inhibition, leading to mucosal barrier disruption and local immune activation [18]. Proton pump inhibitors have been associated with CC, due to their effects on gastric pH and microbiota [18]. Other agents, including selective serotonin reuptake inhibitors, some statins, beta‐blockers, and angiotensin‐converting enzyme inhibitors, may be implicated [3, 4, 18]. Evidence establishing a direct causal relationship is lacking; many of these drugs list diarrhea as a side effect, but in some cases, discontinuation of the suspected medication led to clinical improvement [3, 4, 18].

Finally, a recent case of CC was reported in a patient treated with secukinumab, an anti‐IL‐17A monoclonal antibody, for psoriatic arthritis [9]. The role of IL‐17 inhibitors in the development of CC remains unknown. IL‐17 plays a central role in maintaining intestinal barrier integrity and regulating mucosal immune responses. Its inhibition may promote increased intestinal permeability and dysregulation of local inflammatory pathways, but current data remain contradictory. Thus, although no direct causal link has been demonstrated to date, clinicians should remain vigilant for the emergence of gastrointestinal symptoms in patients treated with IL‐17 inhibitors [9].

### 3.5 Therapeutic Management

The primary goal of treatment for CC is to control symptoms and reverse histological changes in the colonic mucosa to achieve clinical remission and improve patients' QOL [1, 13, 15]. Initially, it is essential to identify and eliminate aggravating factors [3, 17, 18].

For symptomatic management of diarrhea, loperamide represents first‐line therapy [1, 13]. Bismuth subsalicylate also leads to remission in the majority of patients, although its use is limited by potential nephrotoxicity and neurotoxicity with prolonged administration [1, 13].

Local and systemic anti‐inflammatory agents play a central role in managing CC. Sulfasalazine has been used empirically based on treatment models for classic IBDs and induces clinical improvement in 40%–50% of patients. Systemic corticosteroids are effective but often require high doses, with a significant risk of adverse effects and relapse upon discontinuation; therefore, they are not recommended as first‐line therapy [1, 13, 15]. Budesonide is the treatment of choice for inducing and maintaining remission: 80%–88% of patients achieve clinical remission, with rapid improvement in QOL [1, 15]. Moreover, budesonide has a favorable safety profile, even during prolonged use, although precautions regarding osteoporosis and cumulative exposure are advised [1, 13, 15].

In patients with refractory disease, immunomodulators represent a therapeutic option [1, 15]. Azathioprine and 6‐mercaptopurine have shown efficacy, unlike methotrexate. For severe or resistant cases, biologic agents may be considered. TNF‐α inhibitors (infliximab or adalimumab) have achieved clinical or histological remission in some patients, while vedolizumab, through selective inhibition of α4β7 integrin, limits T‐lymphocyte migration into the gut, providing localized efficacy with a favorable safety profile, particularly in elderly patients [1, 13, 15].

Surgery remains an exceptional option. Intestinal diversion via ileostomy can induce histological remission. However, restoration of intestinal continuity generally results in the reappearance of the collagen band [1, 13, 15].

### 3.6 Associated Autoimmune Disorders

Unlike other IBDs, CC is characterized by the rarity of extraintestinal manifestations but is strongly associated with systemic autoimmune conditions. Indeed, 20%–60% of patients present with at least one additional autoimmune disorder, supporting the hypothesis of an underlying immune‐mediated mechanism [5, 6, 7]. The most frequently reported associations are autoimmune thyroid diseases, rheumatoid arthritis, Sjögren's syndrome, and celiac disease. Other reported conditions include discoid lupus, systemic lupus erythematosus, myasthenia gravis, scleroderma, vitiligo, polymyalgia rheumatica, and mixed connective tissue diseases [5, 6, 7]. Immunologically, ANAs are more frequently observed than in healthy populations, suggesting systemic immune activation [6, 7].

### 3.7 Arthropathies Associated With CC

CC‐associated arthropathy is frequently underdiagnosed. Since the first description of arthritis associated with CC by Erlendsson et al. in 1983 [19], a heterogeneous spectrum of joint involvement has been reported [10, 11, 12, 20, 21]. Published cases remain rare but consistent, highlighting that CC should be considered among the growing list of “enteropathic” arthropathies [11, 12, 20, 21]. Two major clinical patterns emerge in the literature: peripheral seronegative arthropathies, generally nonerosive and nondeforming, and true SpA, which is less frequent but well‐documented [12, 20, 21].

Peripheral arthritis associated with CC most often manifests as oligoarthritis or polyarthritis affecting large joints, often asymmetrically [10, 11, 12, 20, 21]. These are typically seronegative, nondeforming, nonerosive, and generally respond well to NSAIDs, sulfasalazine, or low‐dose oral corticosteroids [10, 11, 20]. Most of these synovitides follow a relapsing course with resolution after intestinal flare remission or CC treatment, although prolonged and rarely destructive forms have been described [11, 12]. The reported frequency of peripheral synovitis is approximately 7%–10%, comparable to that observed in UC but lower than in CD [8, 21].

SpA associated with CC is uncommon. To date, few cases fulfilling the ESSG (European Spondylarthropathy Study Group criteria) classification criteria for SpA have been published; these forms are characterized by combined axial and peripheral involvement, a notable proportion of clinical or radiographic sacroiliitis, and an inflammatory spinal pattern that can be clinically indistinguishable from ankylosing spondylitis [12, 20, 21]. HLA‐B27 is found in approximately half of reported CC‐SpA cases [20, 21]. Dactylitis and enthesopathies have been observed in 25% of patients, and some cases progressed to chronicity with ankylosis and erosive changes despite control of colitis [12, 21].

From a pathophysiological perspective, the CC‐arthropathy association plausibly fits an enteric model: an immune‐mediated condition may simultaneously affect the colonic mucosa and the musculoskeletal system [5]. Digestive lesions observed in CC sometimes coexist with synovial infiltrates rich in lymphocytes and plasma cells. The most frequently proposed hypothesis involves increased intestinal permeability, allowing luminal antigens to stimulate local T‐ and B‐lymphocytes, which subsequently migrate to the synovium or skin and trigger inflammation [5].

Clinically, several points are crucial for rheumatologists. The occurrence of chronic diarrhea in a patient with seronegative arthritis or axial signs should raise suspicion of MC and prompt discussion of colonic biopsies even in the absence of macroscopic endoscopic abnormalities [2, 13, 16]. Conversely, in patients with a diagnosis of CC, active screening for musculoskeletal symptoms and rheumatological evaluation are warranted [10, 11, 12, 20, 21]. Therapeutic conflicts should be noted: NSAIDs, effective for arthritis, may exacerbate or even induce MC and should be used cautiously [18]; sulfasalazine is often a practical choice, as it can relieve peripheral arthritis while potentially benefiting the colonic mucosa [1]. For refractory cases, TNF‐α inhibitors may control both joint and colonic involvement, although clinical experience remains limited [10, 11, 12, 20, 21]. In contrast, IL‐17 inhibitors require caution due to reports of new IBD diagnoses or gastrointestinal flares [9].

## 4 Conclusion

Although primarily characterized by chronic watery diarrhea and abdominal pain, CC may be accompanied by seronegative peripheral arthropathies or axial/peripheral SpA, which are often unrecognized and underdiagnosed. Shared immune‐mediated mechanisms between the colonic mucosa and joints suggest a common pathogenic link, further supported by the frequent association with other autoimmune diseases. Close collaboration between rheumatologists and gastroenterologists is essential for early diagnosis and integrated management.

## Author Contributions

**Charlotte Bouvy:** conceptualization, writing – original draft, writing – review and editing. **Abeline Kapuczinski:** supervision. **Sophiane Ibrahimi:** supervision. **Marc Léon:** supervision. **Nathalie Demeulenaere:** supervision.

## Funding

The authors have nothing to report.

## Consent

Published with written consent of the patient.

## Conflicts of Interest

The authors declare no conflicts of interest.

## Data Availability

Data sharing not applicable to this article as no datasets were generated or analysed during the current study.
